# The effect of seasonality on reproductive outcome of patients undergoing intracytoplasmic sperm injection: A descriptive cross-sectional study

**DOI:** 10.18502/ijrm.v13i11.7967

**Published:** 2020-11-22

**Authors:** Marzieh Mehrafza, Maryam Asgharnia, Azadeh Raoufi, Elmira Hosseinzadeh, Sajedeh Samadnia, Zahra Atrkar Roushan

**Affiliations:** ^1^Mehr Fertility Research Center, Guilan University of Medical Sciences, Rasht, Iran.; ^2^Reproductive Health Research Center, Department of Obstetrics and Gynecology, Alzahra Hospital, School of Medicine, Guilan University of Medical Sciences, Rasht, Iran.

**Keywords:** Intracytoplasmic sperm injection, Seasons, Pregnancy outcome.

## Abstract

**Background:**

There is conflicting evidence regarding the impact of season on the assisted reproductive technology outcome.

**Objective:**

To retrospectively compare three year outcome of women undergoing their first intracytoplasmic sperm injection cycle, across seasons.

**Materials and Methods:**

In this descriptive cross-sectional study, 3,670 women who underwent their first intracytoplasmic sperm injection cycle in Mehr Medical Institute, Rasht, Iran between April 2010 and May 2014 were studied. Women were divided into four groups according to the day of oocyte retrival as: spring (n = 808), summer (n = 994), autumn (n = 1066), and winter (n = 802). Basal and stimulation charecteristics were compared among groups.

**Results:**

While sperm concentration and motility were significantly lower during summer, the total number of retrieved and metaphase II oocytes were significantly higher (p = 0.0001, p = 0.0001, p = 0.004, p = 0.02, respectively). Fertilization rate were significantly higher during autumn (p = 0.0001). Also, the number of high- quality transferred embryos were significantly higher during summer and winter (p = 0.03). A similar pattern was observed in implantation rate and pregnancy over the four seasons.

**Conclusion:**

Despite the fact that intracytoplasmic sperm injection minimize the seasonal effect on pregnancy outcome, changes in pregnancy rate still occur among different seasons without particular pattern. It seems that performing assisted reproductive technology procedures in a particular season should be considered as an effective factor.

## 1. Introduction

The impact of seasonality on mammals reproduction has been proven by many studies (1-3). Moreover, numerous studies have suggested that human reproduction can be influenced by variation in seasonal factors including photoperiods and temperature (4). Semen parameters, oocyte quality, and endometrial receptivity are the main factors influenced by seasonality (5, 6). Hormone production and some physiological process including body temperature and rest-activity cycle have circadian rhythms that are in accordance with daily light-dark cycle. Photoperiodism triggers melatonin secretion, resulting changes in pituitary-ovarian axis hormones (7). Although, most seasonal variation in ovulation rate and semen parameters is compensated by intracytoplasmic sperm injection (ICSI) procedures, it provides a good model to evaluate the effect of seasons on human reproduction. Moreover, there are conflicting bodies of evidence on the seasonal variation of assisted reproductive technology (ART) outcome. While some studies suggested the effects of seasonality on the in vitro fertilization (IVF) outcome, the others failed to confirm any possible effects (8, 9).

Considering these controversial results, we retrospectively compared the three-year outcome of patients undergoing their first ICSI cycle, across four seasons.

## 2. Materials and Methods

### Study design

This descriptive cross-sectional study was conducted on women who underwent their first ICSI cycle between April 2010 and May 2014 at Mehr Medical Institute, Rasht, Iran. Canceled cycles for no retrieved oocytes or no embryos suitable for transfer were excluded from the analysis. A total of 4,215 women were evaluated, of which 3,670 women were included in the study. Based on the day of oocyte retrieval, women were grouped into four seasons: spring (n = 808), summer (n = 994), autumn (n = 1066), and winter (n = 802). Semen quality, total number of retrieved and metaphase II oocytes, embryo quality, the rate of fertilization, implantation, and pregnancy were compared among groups.

### Ovarian stimulation

Gonadotropin releasing hormone (GnRH) analogues were used for pituitary suppression. In GnRH agonist cycles, decapeptyl (1.25 mg, Ferring, Germany) was adminstrared on the 21${}^{\mathrm{st}}$ day of previous cycle. In patients underwent GnRH antagonist, cetrotide 0.25 mg (Merck-Serono, Switzerland) were adminstrated daily when the leading follicle reached 14mm in diameter. Ovarian stimulation was done with recombinant FSH (rFSH, Gonal-F, Serono, Germany) or human menopausal gonadotropin (hMG, Menopur, Fering, Germany) adjusted to the ovarian response. Transvaginal ultrasonography and estradiol measurments were used for follicle development. After triggering final oocyte maturation, oocyte pick up were done. The ICSI was performed after the denudation of oocyt-cumulus complexes. Next, three to five days following the ICSI procedures, up to three embryos were transferred. The fertilization rate was calculated by dividing the number of the two pronucli by the total number of metaphase II oocytes. The implantation rate was defined as the ratio between the number of gestational sac and embryo transferred. Clinical pregnancy was proved by the presense of gestational sac with at seven weeks of pregnancy.

Moreover, the classification of embryo quality was based on the following criteria:

• Excellent quality (A)

Day 3: 6-8 even-sized blastomeres with ≤ 10% fragmentation.

Day 5: Blastocysts with an expanded blastocoel, thin zona pellucida, and clear inner cell mass (ICM) with a large number of cells that are tightly interconnected together with a large number of small cells that form the trophectoderm layer.

• Good quality (B)

Day 3: 6-8 blastomeres of both equal and unequal size with 10-20% fragmentation.

Day 5: Blastocysts with an expanded blastocoel, loose ICM, and trophectoderm.

• Poor quality (C)

The number of low and unequal size blastomeres with a fragmentation > 20%. Blastocysts with small blastocoel, lack of ICM, and non-coherent trophectoderm layer.

### Ethical consideration

All participants gave written informed consent for the use of their medical data in research. The present study was approved by the ethics committee of the Guilan University of Medical Sciences (code: IR.GUMS.REC.1394.168).

### Statistical analysis

Data were presented as mean ± standard deviation (SD) and percentage (%). Continuous and categorical variables were analyzed using analysis of variance test (ANOVA) and Chi-square goodness of fit tests, respectively. In addition, as a follow-up to the ANOVA test, Tukey's HSD post-hoc test was used. Statistical analysis was done using the statistical package for the social sciences version 21 (SPSS Inc. Chicago, IL, USA). To control the impact of confounding variables on pregnancy outcome, logistic regression analysis was performed. P < 0.05 was considered as statistically significant.

## 3. Results

The mean age and BMI of women was 34.99 ± 6.55 yr and 26.93 ± 4.69 kg/m${}^{2}$, respectively. The causes of infertility were classified into four groups: male infertility (37.15%), female infertility (44.15%), both female and male infertility (15%), and unexplained infertility (8.65%). All Clinical characteristics of patients were presented in table I. Sperm concentration and motility, number of retrieved and metaphase II oocytes were significantly different across seasons. The difference in sperm morphology was comparable among groups. The fertilization rates was significantly higher in autumn and summer than winter and spring. While no significant difference was observed in the number of high-quality embryos among the four groups), the difference in the numbers of high-quality transferred embryos were significant. In addition, there was a significant difference in the rate of implantation and pregnancy across four seasons. After controlling the impact of the number of high-quality transferred embryos, the logistic regression analysis also described the association between seasonality and pregnancy outcome (Table II).

**Table 1:** Clinical characteristics of patients


**Variables**	**Spring**	**Summer**	**Autumn**	**Winter**	**P-value**
Sperm concentration (×10${}^{6}$)*	41.38 ± 20.25	37.49 ± 20.15	38.20 ± 20.43	42.78 ± 21.34	0.0001${}^{a}$
Sperm motility (%)*	64.32 ± 18.14	61.37 ± 18.17	62.04 ± 18.34	65.29 ± 18.34	0.0001${}^{a}$
Sperm morphology (%)*	13.30 ± 8.23	13.28 ± 8.58	13.31 ± 8.36	14.29 ± 8.2	0.09${}^{a}$
Total number of retrieved oocytes*	9.64 ± 6.34	10.97 ± 7.56	9.99 ± 7.09	10.11 ± 6.59	0.004${}^{a}$
Total number of metaphase II oocytes*	7.76 ± 5.58	8.75 ± 6.31	8 ± 5.74	8.17 ± 5.81	0.02${}^{a}$
Fertilization rate (%)**	3425/5493 (62)	5400/8115 (67)	5481/7241 (72)	3653/5922 (62)	0.0001${}^{a}$
Number of high-quality embryos*	2.22 ± 2.7	2.45 ± 2.5	2.42 ± 2.5	2.49 ± 2.7	0.23${}^{a}$
Number of high-quality transferred embryos*	1.57 ± 1.4	1.74 ± 1.4	1.72 ± 1.4	1.79 ± 1.4	0.03${}^{a}$
Implantation rate (%)**	316/1792 (18)	527/2552 (21)	428/2571 (17)	417/1979 (22)	0.0001${}^{a}$
Clinical pregnancy rate (%)**	231/716 (32.3)	380/933 (40.7)	331/998 (33.2)	300/737 (40.7)	0.0001${}^{b}$
Data presented as * Mean ± SD and ** percentage; ${}^{a}$ANOVA test and ${}^{b}$ Chi-square test					

**Table 2:** The cruds and adjusted odds ratios of clinical pregnancy for the number of high-quality transferred embryos


	**P-value ${}^{a}$**	**Crude OR**	**95% CI**	**P-value${}^{a}$**	**Adjusted OR**	**95% CI**
**Winter **	0.002	1.41	1.14, 1.75	0.04	1.28	1.01, 1.62
**Spring**	0.85	0.98	0.81, 1.20	0.64	0.95	0.77, 1.18
**Summer **	0.003	1.35	1.11, 1.65	0.01	1.32	1.06, 1.64
**Autumn **	0.000	0.66	0.63,0.70	0.000	0.66	0.63,0.70
**No. of high-quality transferred embryos**	0.000	1.49	- -	- -
OR: Odds ratio; CI: Confidence interval; ${}^{a}$Logistic regression analysis						

## 4. Discussion

The results of present study indicated that changes in pregnancy rate occur among different seasons without particular pattern. It seems that performing assisted reproductive technology procedures in a particular season should be considered as an effective factor.

After a Careful study of the literature on the seasonal variations in the ART cycles outcome, the extent of variation between these studies is remarkable. In contrast with study which cannot confirm the seasonal changes of ART outcome (9), there are some reports from different countries that have suggested the relation between seasonality and IVF results. However, there was no consensus on the superiority of one season for optimal pregnancy outcome. A study on 2,709 IVF cycles has reported an improved pregnancy rate during summer IVF cycles (10), whereas, the study by Braga and colleagues on ICSI patients demonstrated higher fertilization rate during spring cycles (11). Also, there are studies that could not specify a pattern for ART outcome during different seasons. Gindes and co-workers (12) evaluated the seasonal fluctuation of 3,522 ART cycles pregnancy rate and concluded a random seasonal pattern. A study by Liu and co-workers (13) aimed to evaluate the relationship between seasonal changes and live births and reported that seasonal changes did not produce significant differences in IVF results with fresh and frozen transfer. Also, a study by Xiao and co-authors (14) showed that seasonal variations have little effect on IVF outcomes and so these treatments can be administered at any season.

The effects of seasonality on reproduction system are mediated by photoperiods and temperature. Light-induced retinal photoreceptors regulate melatonin secretion from pinealocytes. The effect of melatonin on GnRH-induced serum FSH and LH level can demonstrate seasonal variation in reproductive outcome. Circadian dysrhythmia or sleep disturbance can lead to changes in the levels of LH, FSH, and prolactin, which in turn affect the success rate of fertility treatments (15).

The progression of primordial follicles into preovulatory follicles is a continuous process lasting several months, which controlled ovarian stimulation takes place only the last three wk of these cycles. Hence, the assessment of environmental impacts on oocyte development became difficult. In the present study, the ovarian response measured by number of retrieved and metaphase II oocytes were significantly higher in summer and the oocyte quality reflected by fertilization potential were significantly higher in autumn and summer than winter and spring. Similarly, Rojansky and colleagues (16) indicated that the fertilization rate and embryo quality was influenced by seasonality. However, Revelli and co-workers (8) could not find a link between fertilization rate and seasons. In a study by Braga and co-authors (11), higher fertilization rate during spring was reported. It seems that higher ovarian response during summer is associated with level of melatonin. The suppressive effect of melatonin on reproductive outcome was first described in pathological conditions like genital tumors (17) and poor reproductive outcome during dark seasons (18).

There are pieces of evidence on the influence of seasonal parameters including temperature and photoperiod on semen parameters (5). However, contrary to these studies, a study aimed at assessing the relationship between seasonal variation and micro dissection testicular sperm extraction success showed no significant relationship between the rate of sperm retrieval and season (19). Whereas, in our study, a significant seasonal fluctuation in sperm concentration and progressive motile sperm with lower level in summer was found. Centalo and colleagues (20) demonstrated lower sperm concentration and motility in summer. According to Reinberg *et al* (21) the maximal levels of estradiol, testosterone, and LH was in autumn which resulted in higher sperm count. Unlike female hemostatic system, limited scrotal thermoregulation resulted in higher temperature sensitivity of semen parameters (22, 23).

In the present study, there was no significant difference in the number of high-quality embryos across four seasons, however, the numbers of high-quality transferred embryos were significantly higher in winter and summer. It can be assumed that higher pregnancy rate during summer and winter could be caused by higher numbers of high-quality transferred embryos. After controlling this confounding variable, the logistic regression analysis also described an association between seasonality and pregnancy outcome. We could not find any reasons for these conflicting results; however, we assumed that it might be associated with low seasonal contrast of north of Iran.

##  Limitation

This study has some limitations which have to be pointed out. First, IVF cycles were not grouped according to the month of ovarian puncture. Second, the level of vitamin D as a factor influenced by seasonality and sunlight exposure was not evaluated in the present study because most of the participants received vitamin D supplementation prior to the ART cycles. Therefore, the seasonal deficiency of vitamin D could not be taken into account. Third, due to the retrospective nature of this study, some confounding factors might have influenced the results. Despite ICSI procedures minimizing the impact of seasonality on pregnancy outcome, changes in pregnancy rate still occur during different seasons, without any particular pattern. In conclusion, it seems that performing ART procedures in a particular season should be considered as an effective factor.

##  Conflict of Interest

The authors declare that there is no conflict of interest in the present study.
